# Supplementary material for: Cover crop and crop residue removal effects on temporal dynamics of soil carbon and nitrogen in a temperate, humid climate
Source: PLoS One. 2020 Jul 9;15(7):e0235665. doi: 10.1371/journal.pone.0235665 (PMC7347225; doi:10.1371/journal.pone.0235665)
Supplement: Attachment — Submitted filename: Response to reviewers R1PLOS One.docx [file pone.0235665.s007.docx]

**Dear Editor-in-Chief,**

**Responses of authors to reviewer’s comments are indicated in blue font.**

1. Comments from the editor:

ABSTRACT

Please note that I revised the abstract before reading the whole manuscript. Additional comments on the abstract after reading the manuscript, if any, can be found at the end of this revision (Additional suggestions)

The abstract is a little bit puzzling. The reader has no clear idea of the setup, and you use a writing style that leave too much aspects on the reader’s perception. I suggest to better describe the setup, especially in the methodological aspects. Just a suggestion as a non-native speaker. Non-native speakers have problems in perceiving the correct meaning of “Temporally”. I suggest to change it.

Authors: Agreed. We have significantly revised the abstract and provided more details about the treatments. Abstract was revised based on this and other reviewer comments. Line 18 “Quantification of seasonal dynamics of soil C and N pools is crucial to understand the land management practices for enhancing agricultural sustainability. In a cover crop (CC) experiment established in 2007 and repeated at an adjacent site in 2008, we evaluated the medium-term impact of CC (no cover crop control (no-CC), oat (*Avena sativa* L.), oilseed radish (OSR, *Raphanus sativus* L. var. *oleoferus* Metzg. Stokes), winter cereal rye (rye, *Secale cereale* L.), and a mixture of OSR+Rye) and crop residue management (residue removed (-R) and residue retained (+R)) on soil C and N dynamics and sequestration. Soil indicators of labile and stable fractions of C and N were determined at seven different time points from 0-15 cm depth during tomato (*Solanum lycopersicum* L.) growing season in 2015 and 2016 (referred to as site-years). As expected, over the tomato growing season in both site-years, organic C (OC) and total N did not change while the indicators of labile C and N fractions changed with greater concentrations observed at 2 weeks after tillage (WAT) and greater treatment differences observed for seven out of eleven indicators at tomato harvest. Therefore, 2WAT (early June) and tomato harvest (early September) are reasonably optimum sampling times for soil C and N attributes. Seasonal variation of labile indicators suggested the potential impact of substrate availability from crop residues on soil C and N cycling. Medium-term CC usage enhanced the concentration of stable and labile pools of C and N. Overall, this study highlights the positive and synergistic influences of CCs and maintaining crop residues in improving soil C and N pools and enhancing soil quality in a temperate humid climate. “

INTRODUCTION

L42-44: unclear how and why

Authors: Clarified in text by inserting and revising lines 55“Winter wheat straw has a high C:N (80:1), thus, leaving winter wheat straw in the field may cause N immobilization with a potential for crop N deficiency. Moreover, soils with crop residue left on the surface are colder and wetter which results in a delayed spring planting. “

L85: please better link it to the previous sentences

Authors: We have significantly revised the introduction based on this and other reviewer comments. These lines were deleted and paragraph was revised as Line 105 “To date, there is a limited knowledge on the potential interactive effects of CCs and crop residue removal on soil C and N dynamics. Additionally, CC and residue removal impacts on soil C and N accumulation are expected to be observed in the medium- to long-term as opposed to the short-term (see reviews by [2,3,11]). Therefore, the medium-term CC experiment used in this study was ideally suited for evaluating the CC- and crop residue-induced effects on C and N dynamics. The main objective of the study was to assess the mechanism of C and N accumulation with CCs and crop residue management (removed (-R) and retained (+R)). We hypothesize that a potential synergistic effect of enhancing crop diversity by including CCs and maintaining crop residues will be observed on soil C and N sequestration. The study is expected to advance our understanding of the integrative effect of CCs and crop residue removal on soil processes involved in C and N cycling.”

L86-90: the aim as it is described is quite confusing. The reader has no idea of the implication of the years, nor it’s needed here. Just say the aim with few, but important, methodological aspects. Were CC and residues management in interaction or not? What does residue management means? If you mean “removal or not”, just declare it. How many tomato growing seasons were studied? L90: with regards to the stabile, this hypothesis contrast with the hypothesis 2 unless you studied a long term, too. And this is unclear.

Authors: Agreed. We have removed years from the objectives. We revised the objectives and hypothesis to provide clarification. The objectives and hypotheses were edited based on this and other reviewer comments. Line 111 “The main objective of the study was to assess the mechanism of C and N accumulation with CCs and crop residue management (removed (-R) and retained (+R)). We hypothesize that a potential synergistic effect of enhancing crop diversity by including CCs and maintaining crop residues will be observed on soil C and N sequestration. The study is expected to advance our understanding of the integrative effect of CCs and crop residue removal on soil processes involved in C and N cycling.”

MATERIALS AND METHODS

L94: quiet unclear, the reader mustn’t be obliged to read the others, just recall those setups.

Authors: Agreed. We have revised and reorganized the section “site description and experimental design” for clarity. Line 125 “The medium-term CC experiment, established in 2007 and repeated an adjacent site in 2008 at Ridgetown, Ontario, Canada, was used for studying soil C and N dynamics. Site-year was used to clearly indicate that soil and crop sampling was conducted from different sites in different years. Soil characteristics are described in Table 1. Since the initiation of the CC experiment in 2007 and 2008, crop rotation consisted of grain and processing vegetable crops, typical of southwestern Ontario (Table 2). As described previously [6,33-36], experimental design consisted of a split-plot arranged as a randomized complete block with four replicates. Summer-planted (July, August, September) annual CC treatments were grown in the main plots since 2007 and 2008. In addition to a no-CC, four CC treatments were evaluated which were oat (*Avena sativa* L.), oilseed radish (OSR) (*Raphanus sativus* L. var. *oleoferus* Metzg. Stokes), winter cereal rye (rye, *Secale cereale* L.), and a mixture of OSR+Rye. Cover crops were not planted after grain corn harvest in late October/early November due to the cold temperatures; after soybean harvest, winter wheat was planted instead of CCs. Crop residue management was applied in the split-plots three times since the experiment started at both sites (in 2009 and 2010 after spring wheat harvest, 2011 and 2012 after grain corn harvest, and 2014 and 2015 after winter wheat harvest) in the production system. Thus, from 2007 to 2015 and 2008 to 2016, CCs were planted six times in the crop rotation (Table 2). Cover crop attributes (biomass, C and N concentration) were quantified three times (late October/early November, April, and May) in 2014-15 and 2015-16. “

L95-98: I think this could be efficiently reported in a time-picture

Authors: We agree and have added Table 2 for clarity. Line 128” Since the initiation of the CC experiment in 2007 and 2008, crop rotation consisted of grain and processing vegetable crops, typical of southwestern Ontario (Table 2).”

**Table 2. In a medium-term cover crop experiment, crop rotation and crop residue management from 2007 to 2015 (site-year 2015) and 2008 to 2016 (site-year 2016)**

| Site-year 2015 | Site-year 2016 | Main crop | Fall planting |
| --- | --- | --- | --- |
| 2007 | 2008 | Processing pea | Cover crops |
| 2008 | 2009 | Processing sweet corn | Cover crops |
| 2009 | 2010 | Spring wheat | Cover crops |
| 2010 | 2011 | Processing tomato | Cover crops |
| 2011 | 2012 | Grain corn -stover removal | None |
| 2012 | 2013 | Processing squash | Cover crops |
| 2013 | 2014 | Soybean | Winter wheat |
| 2014 | 2015 | Winter wheat -straw removal | Cover crops |
| 2015 | 2016 | Processing tomato | Cover crops |

L166: protected LSD is marginally ok for your setup. Since the data are balanced, you may have had few or no problems of difference estimation. For your further work, I suggest to use pdifferences of the LSmeans, eventually corrected by Tukey Kramer depending on the balancing of the setup.

Authors: Agreed. Thank you. Due to a balanced dataset, no problems were encountered in estimating mean differences with the protected LSD test. Therefore, we did not change the mean adjustment method in this manuscript.

RESULTS

L202: as an editor, I am not interested in the shape you give to the figure, but I suggest to ass year as a random factor or, if retaining this analysis, split the years in the figures, with one year in a side and the other in the other side. Treatments are within years, not its contrary. The results of the analysis you are providing are unclear. The reader wants to know the mean effect of the treatments across years. Lines recall a trend, but this is not the case.

Authors: Agreed. We revised Fig 2 based on your comments. We also revised the results for clarity. Line 261 “Over 6-yrs from fall 2007 to fall 2014, average annual CC biomass across all CC treatments was 3220±115 kg ha^-1^, C was 1280±46.2 kg C ha^-1^, and N was 78±3.47 kg N ha^-1^ (Fig 2). Similarly, from fall 2008 to fall 2015, average annual CC biomass across all CC treatments was 3520±116 kg ha^-1^, C was 1290±50.7 kg C ha^-1^, and N was 97.7±4.15 kg N ha^-1^ (Fig 2).”

L222: providing the p<0.05 in bold helps in reading

Authors: Agreed and edited Table 3 and 4. Added a footnote for clarity. Line 295 and Line 303 “Bold font indicates statistical significance at *P*<0.05”

L250: this pc must be transformed in order to be more readable. Please change colors, sizes, and when applicable y-axes. Do the same for fig. 4

Authors: Agreed. We revised Figs 3, 4, 5, and 6 by color coding different treatments, increasing font size, and adding the time of tillage, tomato transplanting and harvest.

DISCUSSION and CONCLUSIONS

L417-418: I suggest to remove this sentence

Authors: Agreed. Sentence deleted

**Reviewers' comments:**
Reviewer 1 comments:

Reviewer #1: This manuscript reports the results of a study aimed at evaluating the short and long term effect on soil C and N indicators of cover crops and crop residues incorporation in the soil. The paper is clearly written and all the sections are appropriately developed. Table are well designed and clear. Unfortunately, the quality of the figure is not adequate at all: they are totally fuzzy, the resolution is too low and they are unintelligible.

Authors: Thank you. We are unsure why the resolution was poor. We have revised the Fig 2 to Fig 6 based on this and other reviewer’s comments. We edited the figures by color coding different treatments, increasing font size, and adding the time of tillage, tomato transplanting and harvest.

According to my opinion the results about the so defined “short term” indicators are poorly discussed in the light of the interaction of the main experimental factors with the crop (tomato) and its growing phases, which may have influenced the responses. In this regards, I would suggest the Authors to analyse/plot the indicators results versus the crop phenological development stages (i.e. BBCH-scale) instead of time (weeks). This approach could help to understand the potential effect of crop growing and its effect, via rhizosphere equilibrium, on C and N short term soil indicators.

Authors: Thank you. We agree that plotting the indicators vs the crop phenological development stages might be helpful in interpreting the study results. But, the main objective of the study was not to assess how these indicators perform at each phenological stage. Because it was not the study goal, we could not be precise enough to indicate these stages in the graphs. We did not target the soil sampling based on crop phenology; therefore, might not be able to specifically mention and discuss the indicator performance at each stage.

To clarify the reviewer’s comment, we have added tomato transplanting and harvesting in the graphs for clarity. Additionally, we have discussed the potential role of crop growth in influencing the dynamics of soil indicators. Line 418 “Labile pools of soil C and N are crucial for soil biology as they act as a nutrient reservoir for soil microbes [57]. Seasonal variation in the labile indicators observed might be attributed to the root exudates released from the tomato roots. Labile C compounds released by the roots, rhizodepositions, are reported to be greater at flowering than planting stage, are incorporated into the microbial biomass; thus, directly impact the microbial community structure and functionality and enhance the concentrations of labile pools of C and N. Several studies have reported that root C and N inputs are largely responsible for influencing the soil biological activity [59,60]. As the crop matures, the concentration of rhizo-deposits decreases but fresh crop residues and mature roots are added to the soil which contribute to the microbial substrates, therefore, increasing the labile fractions of C and N at harvest [61].”

Line 428 “Overall, our observed variation in the concentrations of labile indicators across the growing season and between years might be attributed to the differences in the quantity of CC biomass produced among the tested CCs and the interactions between soil microbes and plant roots.”

However, the main flak of the paper is that, in my opinion, it is not novel enough. The most of the considerations and the conclusions are already known and no new information is reported in the manuscript. For this reason I would not suggest to accept the paper for publication in Plos One.
Authors: We have significantly revised the introduction to indicate the need for this study and the contribution of this study in advancing our knowledge of soil C and N cycling. We have also revised our objectives and hypothesis for clarity. Line 92 “A better understanding of the seasonal dynamics of soil C and N will advance our knowledge for improving agricultural management practices focusing on enhancing agricultural sustainability, soil quality, and climate change mitigation. Thus, assessments of seasonal variability in soil labile indicators are needed.”

Line 105 “To date, there is a limited knowledge on the potential interactive effects of CCs and crop residue removal on soil C and N dynamics. Additionally, CC and residue removal impacts on soil C and N accumulation are expected to be observed in the medium- to long-term as opposed to the short-term (see reviews by [2,3,11]). Therefore, the medium-term CC experiment used in this study was ideally suited for evaluating the CC- and crop residue-induced effects on C and N dynamics. The main objective of the study was to assess the mechanism of C and N accumulation with CCs and crop residue management (removed (-R) and retained (+R)). We hypothesize that a potential synergistic effect of enhancing crop diversity by including CCs and maintaining crop residues will be observed on soil C and N sequestration. The study is expected to advance our understanding of the integrative effect of CCs and crop residue removal on soil processes involved in C and N cycling.”

Reviewer #2: Review PONE-D-19-28936
Cover crop and crop residue removal effects on temporal dynamics of soil carbon and nitrogen in a temperate, humid climate
by Chahal & Van Eerd

Content
The paper describes the impact of 4 cover crops planted 6 years and the use of crop residues 3 times in a field experiment on soil quality indicators associated with the C and N cycles. The field trial was conducted twice, starting in 2007 and 2008. Apart from cumulative effects of cover crops and residue management on soil quality indicators across the live span of the field experiment, the time course in the growth season of tomatoes was measured my multiple sampling to assess short term effects of cover crops.
The authors found, that both, cover crops and crop residues increased soil organic carbon and total nitrogen. As expected, there were no seasonal changes of soil organic carbon and total nitrogen, but label carbon and nitrogen fractions, soil respiration and microbial biomass N and C were affected.
Although the findings are interesting, I have several concerns how the manuscript is presented and written.

General comments
1. The language and writing style is very technical, using a lot of abbreviations.

Authors: To facilitate an easy interpretation of manuscript and avoid confusion, we replaced TN with total N at 34 instances and TIN with total inorganic N at 25 instances. We also significantly revised all the sections of the manuscript for clarity.

The way how the manuscript is written, it is more addressing a specialist readership, and to a less extent a general audience. In addition, the text is often not clear, for example, the abstract is not understandable without reading the full paper. What are time-years, double parenthesis are not easy to read, etc.

Authors: Based on this and other reviewer comments, we have substantially edited the manuscript with a significant revision to the abstract, introduction, methods, and discussion sections for clarity. Site-years has been clarified in the abstract and methods sections. 6 out of 7 sentences with double parenthesis were revised in the manuscript.

2. The text contains some un-logic constructions with use of several terms not in a systematic manner.
Expl. 1: line 83-84: “The effect of soil functions and treatments on seasonal (or short-term) trends and microbial processes suggests the need for short-term assessments of soil C to avoid misinterpretation of treatment effects on the C dynamics.” Soil functions do not effect seasonal trends and microbial processes. Soil functions, eg N cycling is the result of microbial processes and is affected by cover crops.

Authors: Thank you. We agree with this comment. However, based on this and other reviewer comments, we significantly revised the introduction. This sentence was deleted and thus not revised.

Expl. 2: line 427-429: “Overall, compared to no-CC and crop residue removal treatments, CCs and crop residue retention had greater concentrations of soil parameters temporally indicating the impact on soil C inputs and microbial processes have implications on soil quality”. It may read “… the impact of soil C inputs on microbial processes with implications on soil quality”.

Authors: Agreed. Sentence revised based on the suggestion. Line 520 “Overall, compared to no-CC and crop residue removal treatments, CCs and crop residue retention had greater concentrations of soil parameters temporally indicating the impact of soil C inputs on microbial processes with implications on soil quality.”

3. The hypothesis is not original. “We hypothesize that (i) short-term (seasonal) changes will be detected in the labile indicators of soil C and N across the tomato growing season, (ii) no seasonal variability will be detected in the stable indicators of soil C and N, and (iii) compared with the no cover crop control (no-CC) and crop residue removal (-R), CCs and crop residue retention (+R) treatments will have greater concentrations of labile and stable indicators of soil C and N”. We expect that so-called stable indicators like soil organic carbon and total nitrogen in the soil does not change within season, but label fractions are affected. This is textbook knowledge. I suggest focusing on the question, if cover crops and crop residues can maintain soil organic carbon and total nitrogen, and if there are additive effects.

Authors: Agreed. We revised the hypothesis and objectives based on this and other reviewer comments. Line 113 “The main objective of the study was to assess the mechanism of C and N accumulation with CCs and crop residue management (removed (-R) and retained (+R)). We hypothesize that a potential synergistic effect of enhancing crop diversity by including CCs and maintaining crop residues will be observed on soil C and N sequestration. The study is expected to advance our understanding of the integrative effect of CCs and crop residue removal on soil processes involved in C and N cycling.”

Interactions of labile indicators within season should be discussed in view of their ecological and agronomic relevance, correlations are not so meaningful. To conclude that labile fractions are reflecting seasonal changes is trivial.

Authors: Agreed. We have clarified this comment in the text. Lines 418 “Labile pools of soil C and N are crucial for soil biology as they act as a nutrient reservoir for soil microbes [57]. Seasonal variation in the labile indicators observed might be attributed to the root exudates released from the tomato roots. Labile C compounds released by the roots, rhizodepositions, are reported to be greater at flowering than planting stage, are incorporated into the microbial biomass; thus, directly impact the microbial community structure and functionality and enhance the concentrations of labile pools of C and N. Several studies have reported that root C and N inputs are largely responsible for influencing the soil biological activity [59,60]. As the crop matures, the concentration of rhizo-deposits decreases but fresh crop residues and mature roots are added to the soil which contribute to the microbial substrates, therefore, increasing the labile fractions of C and N at harvest [61].”

PS: Instead of labile and stable indicators, I would prefer to refer to indicators for more stable soil parameters, and indicators for more labile soil quality parameters. At least define what are stable and labile indicators.

Authors: Agreed. We have addressed this comment in line 74 “Soil OC and total N change slowly over the long-term and are considered as the indicators of stable fraction of soil C and N, whereas SLAN (Solvita labile amino N), Solvita CO_2_-burst, microbial biomass C and N (MBC and MBN), water extractable organic C and N (WEOC and WEON), wet aggregate stability (WAS), total inorganic N, and cumulative 2d C mineralization (Cmin_2d_), are known to vary over short (seasonal) term, and thus, are the indicators of labile fraction of C and N.”

We have also replaced the stable indicators as indicators of stable fraction of soil C and N at 15 instances and labile indicators as indicators of labile fractions of soil C and N at 21 instances throughout the manuscript for clarity.

4. The claim that sampling 2 weeks after tillage and after harvest are optimum sampling time points needs further statistical analyses, using multivariate statistics. In all, I cannot recommend the manuscript for publication in PLOS ONE as it stands.

Authors: It is possible and would have been one approach for data analysis. But we did an ANOVA to assess the impact of sampling time on the seasonal dynamics of soil C and N. The tables 3 and 4 and supplemental tables S3 and S4 indicated the statistical significance of C and N indicators over time and provided the mean values of indicators at each sampling time, respectively. A multivariate analysis would have essentially ranked the sampling times with greatest to lowest value of each soil C and N indicator, the analysis would have to be done for each indicator and would have not provided any additional information. The ANOVA analysis employed in the manuscript with sampling time as a fixed effect adequately assessed each soil C and N indicator at each time point and provided treatment differences. Therefore, we did not include the multivariate statistics in the manuscript. We revised Line 461 for clarity.

Line 461 “Greater concentration of soil indicators with CC than no-CC at tomato harvest might be a reflection of a build up of stable and labile pools of C and N due to the cumulative effects of 6-yrs of CCing. Therefore, June and/or September in a crop growing season is a reasonable time for soil sampling to evaluate soil C and N dynamics and overall soil quality.”

Detailed comments
L 61: Microbial activity is not a soil process

Authors: Agreed. Deleted in the sentence Line 82“Microbial activity, is a key factor affecting the cycling of labile pools of C [26] and N.”

L107: The field trial should be described briefly

Authors: Agreed. Based on this and other reviewer’s comment, we have revised the site description and experimental design. Lines 125“The medium-term CC experiment, established in 2007 and repeated an adjacent site in 2008 at Ridgetown, Ontario, Canada, was used for studying soil C and N dynamics. Site-year was used to clearly indicate that soil and crop sampling was conducted from different sites in different years. Soil characteristics are described in Table 1. Since the initiation of the CC experiment in 2007 and 2008, crop rotation consisted of grain and processing vegetable crops, typical of southwestern Ontario (Table 2). As described previously [6,33-36], experimental design consisted of a split-plot arranged as a randomized complete block with four replicates. Summer-planted (July, August, September) annual CC treatments were grown in the main plots since 2007 and 2008. In addition to a no-CC, four CC treatments were evaluated which were oat (*Avena sativa* L.), oilseed radish (OSR) (*Raphanus sativus* L. var. *oleoferus* Metzg. Stokes), winter cereal rye (rye, *Secale cereale* L.), and a mixture of OSR+Rye. Cover crops were not planted after grain corn harvest in late October/early November due to the cold temperatures; after soybean harvest, winter wheat was planted instead of CCs. Crop residue management was applied in the split-plots three times since the experiment started at both sites (in 2009 and 2010 after spring wheat harvest, 2011 and 2012 after grain corn harvest, and 2014 and 2015 after winter wheat harvest) in the production system. Thus, from 2007 to 2015 and 2008 to 2016, CCs were planted six times in the crop rotation (Table 2). Cover crop attributes (biomass, C and N concentration) were quantified three times (late October/early November, April, and May) in 2014-15 and 2015-16. “

L117: Were biomass N concentrations also determined?

Authors: Yes. Added “N concentration” in the text. Line 149 “Cover crop attributes (biomass, C and N concentration) were quantified three times (late October/early November, April, and May) in 2014-15 and 2015-16.”

L120: What is a site-year?

Authors: We provided the clarification at line 126 “Site-year was used to clearly indicate that soil and crop sampling was conducted from different sites in different years.”

L121: Was tillage connected to the incorporation of the cover crops?

Authors: Yes. Clarified in the text. Line 169 “Soil was tilled on May 19, 2015 and May 20, 2016 to incorporate CC residues and fertilizers and to prepare for tomato transplanting.”

L168: What is a mean maximum value?
Authors: We clarified in the text by deleting the word “mean”. Line 233 “data were expressed as a ratio of the average indicator value for each CC treatment and the maximum value of the respective attribute, to facilitate the visual interpretation of the impact of management on soil parameters.”
L173: Growing conditions: were tomato plants irrigated? Should it read growth season?

Authors: Clarified in Line 171 “No irrigation was applied to tomato crop.” Section heading changed to “tomato growing season”

L258ff: You may only refer to differences, which are significant

Authors: Agreed. Sentence revised. Line 331 “Retaining crop residues in the field resulted in greater concentrations of WAS, Cmin_2d_, Solvita CO_2_-burst, and total N in site-year 2015, and WAS, total inorganic N, and WEOC in site-year 2016 than removing residues (S3 and S4 Tables).”

L298-L315: Extremely long text just to describe correlations. I suggest to include a table in the supplement, and to show most important findings as graph in the main part.

Authors: Agreed. We revised the lines 373-392 and prepared Tables S5 and S6 depicting the correlation coefficients between soil indicators. In the tables, the significant correlations were represented by adding an asterisk (*) next to the coefficient. The tables provided a complete information of the relationship between indicators. Therefore, we did not include a graph representing the correlations in the revised manuscript.

L322: Difference in CC biomass and eventually C and N content. Probably? Reasoning?

Authors: We revised the sentence to provide clarity Line 399 “This difference in fall CC biomass (and C and N content) over the study duration was due to timing of main crop harvested (i.e. fresh processing pea in late July and processing tomato in early Sept) and consequently the length of the CC growing season.”

L334: N inputs were 78 to 97 kg ha-1. This is N taken up from soil, no new N inputs
Authors: Sentence revised for clarity. Line 410 “. In our medium-term experiment, average annual CC above-ground C inputs were 1280 to 1380 kg C ha^-1^ and CC above-ground N inputs were 78 to 97 kg N ha^-1^, which contributed to a significant increase in soil OC and total N.”

L345: Is the method to measure respiration precise enough to discern between cover crops? Did you once apply a standard method for comparison?

Authors: Respiration was measured using the standard substrate induced NaOH trap method and the protocol outlined in Canadian Society of Soil Science manual by Carter and Gregorich (2008) was followed. Line 196 “For evaluating Cmin_2d_, substrate-induced respiration was conducted with an addition of glucose (1.6 mg C g^-1^ dry soil) using an alkali trap method with 60 g dry soil [35,42].” Previous study conducted by Ouellette et al. (2016) at the same experimental site evaluated the impact of cover crop treatments on soil respiration using the same standard NaOH method and results were consistent with our study. Moreover, we conducted correlations for soil respiration measured by standard NaOH trap method and commercial soil health test (Solvita CO2-burst) and found a significant positive correlation (Table S5).

L 359ff: To justify that sampling 2 weeks after tillage and after harvest is recommendable to discern short- and medium-term effects of cover crops, you may apply multivariate statistics.

Authors: It is possible and would have been one approach for data analysis. But we did an ANOVA to assess the impact of sampling time on the seasonal dynamics of soil C and N. The tables 3 and 4 and supplemental tables S3 and S4 indicated the statistical significance of C and N indicators over time and provided the mean values of indicators at each sampling time, respectively. A multivariate analysis would have essentially ranked the sampling times with greatest to lowest value of each soil C and N indicator, the analysis would have to be done for each indicator and would have not provided any additional information. The ANOVA analysis employed in the manuscript with sampling time as a fixed effect adequately assessed each soil C and N indicators at each time point and provided treatment differences. Therefore, we did not include the multivariate statistics in the manuscript. We revised Line 461 for clarity.

Line 461 “Greater concentration of soil indicators with CC than no-CC at tomato harvest might be a reflection of a build up of stable and labile pools of C and N due to the cumulative effects of 6-yrs of CCing. Therefore, June and/or September in a crop growing season is a reasonable time for soil sampling to evaluate soil C and N dynamics and overall soil quality.”

L378: What do you mean by: “Peak period of tomato growth initiates in June and greater
indicator concentration during June might have increased the nutrient availability to tomato roots and hence increased crop yield». What is the meaning of this sentence? You did not present yields, and these relations are extremely speculative.

Authors: Agreed. Paragraph deleted.

L387: You did not find a increase of respiration by cover crops.

Authors: Our results indicate greater soil respiration with cover crops than no cover crop control. Additionally, Solvita CO_2_-burst is a commercial soil health test which measures soil respiration. Greater values of evolved CO_2_-C from soil were detected with cover crops than control in both site-years (Table S3 and S4). We revised the sentence for clarity. Line 478 “Similarly, several studies have reported the increases in MBC, MBN, and soil respiration (evolved CO_2_) with CCs [74-77].

L407: Root exudates of CC may also be mentioned to positively affect stable and labile soil quality indicators.

Authors: Agreed. Sentence revised. Line 498 “. Thus, future research elucidating the mechanism of CC (above- and below- ground biomass and root exudates) and crop residue effects on soil C and N cycling and storage is needed.”

L420: TN is not a short-term indicator

Authors: Thank you. Deleted total N

L427-L429: Far from clear

Authors: Sentence revised for clarity. Line 520“Overall, compared to no-CC and crop residue removal treatments, CCs and crop residue retention had greater concentrations of soil parameters temporally indicating the impact of soil C inputs on microbial processes with implications on soil quality.”

L433ff: These conclusions are not based on your results, but rather general.
Authors: Agreed. We revised the sentence to clarify. Line 524 “Additionally, our results demonstrated the role of CCs in increasing OC and total N concentration after 6-yrs of CCing, and highlighted the potential of CCs in sequestering soil C and N with implications on mitigating climate change.”

Tab2 and 3: I suggest to put these tables in the annex

Authors: We do not agree with this comment. Tables 3 and 4 in the revised manuscript represent the statistical effect of time and its interaction with the cover crops and crop residue on soil C and N parameters. Assessing the seasonal variation of soil C and N due to CCs and crop residue is a major goal of the manuscript. Therefore, we did not move the tables 3 and 4 to the annex/supplementary information. In the supplementary information, we have Tables S3 and S4 which provide the treatment (cover crops, crop residue, and sampling time) means for soil C and N indicators.
