# Supplementary material for: Cover crop and crop residue removal effects on temporal dynamics of soil carbon and nitrogen in a temperate, humid climate
Source: PLoS One. 2020 Jul 9;15(7):e0235665. doi: 10.1371/journal.pone.0235665 (PMC7347225; doi:10.1371/journal.pone.0235665)
Supplement: Attachment — Submitted filename: Revised manuscript R1 (clean copy)_ED comments.docx [file pone.0235665.s008.docx]

**Cover crop and crop residue removal effects on temporal dynamics of soil carbon and nitrogen in a temperate, humid climate**

Inderjot Chahal and Laura L. Van Eerd*

School of Environmental Sciences, University of Guelph, Ridgetown Campus, Ridgetown, Ontario, Canada

*Corresponding author

Email: [lvaneerd@uoguelph.ca](mailto:lvaneerd@uoguelph.ca) (LVE)

# **Abstract**

Quantification of seasonal dynamics of soil C and N pools is crucial to understand the land management practices for enhancing agricultural sustainability. In a cover crop (CC) experiment established in 2007 and repeated at an adjacent site in 2008, we evaluated the medium-term impact of CC (no cover crop control (no-CC), oat (*Avena sativa* L.), oilseed radish (OSR, *Raphanus sativus* L. var. *oleoferus* Metzg. Stokes), winter cereal rye (rye, *Secale cereale* L.), and a mixture of OSR+Rye) and crop residue management (residue removed (-R) and residue retained (+R)) on soil C and N dynamics and sequestration. Soil indicators of labile and stable fractions of C and N were determined at seven different time points from 0-15 cm depth during tomato (*Solanum lycopersicum* L.) growing season in 2015 and 2016 (referred to as site-years). As expected, over the tomato growing season in both site-years, organic C (OC) and total N did not change while the indicators of labile C and N fractions changed with greater concentrations observed at 2 weeks after tillage (WAT) and greater treatment differences observed for seven out of eleven indicators at tomato harvest. Therefore, 2WAT (early June) and tomato harvest (early September) are reasonably optimum sampling times for soil C and N attributes. Seasonal variation of labile indicators suggested the potential impact of substrate availability from crop residues on soil C and N cycling. Medium-term CC usage enhanced the concentration of stable and labile pools of C and N. Overall, this study highlights the positive and synergistic influences of CCs and maintaining crop residues in improving soil C and N pools and enhancing soil quality in a temperate humid climate.

*Abbreviations:*

CC, cover crop; Cmin_2d_, cumulative 2d soil C mineralization; MBC, microbial biomass C; MBN, microbial biomass N; no-CC, no cover crop control; OSR, oilseed radish; OSR+Rye, mixture of oilseed radish and winter cereal rye; SLAN, Solvita labile amino N; OC, organic C; WAT, weeks after tillage; WAS, wet aggregate stability; WEOC, water extractable organic C; WEON, water extractable organic N; -R, crop residue removed; +R, crop residue retained.

# **Introduction**

Winter wheat (*Triticum aestivum* L.) followed by processing tomato (*Solanum lycopersicum* L.) is a common vegetable crop rotation in Ontario, Canada, and Midwest USA. Residue management of winter wheat is important as it may influence the microbial processes, N availability, and C dynamics in the following season crop. Winter wheat straw has a high C:N (80:1), thus, leaving winter wheat straw in the field may cause N immobilization with a potential for crop N deficiency. Moreover, soils with crop residue left on the surface are colder and wetter which results in a delayed spring planting. Therefore, to avoid this, growers tend to remove winter wheat straw from the field after grain harvest. Crop residue, however, is a source of C and N for soil macro and micro fauna. Crop residue removal (-R) may have some negative implications on long-term soil productivity and quality [1,2] by reducing soil organic C (OC) and total N levels [3,4], reducing soil microbial activity [5], and increasing wind and water erosion leading to loss of nutrients [2]. However, inclusion of cover crops (CC) after crop residue removal may be an effective management strategy to offset the expected reductions in soil C inputs and soil quality [2].

Several benefits to agroecosystems have been observed with adoption of CC in crop rotations [6-9]. Improvements in soil physical [10-12], biological [2], and chemical [13,14] soil quality indicators are observed with CCs, which are highly dependent on the quantity and quality of CC biomass input to the soil [15]. Cover crops also maintain soil C and N levels by sequestering C and N from atmosphere and mitigating C and N losses [16]; thus, might increase N availability to the subsequent crop [16]. However, timing of N release from the CC residues is a critical factor, which is dependent on the CC C:N, lignin and cellulose concentrations of CC [17,18], and timing of CC termination and residue incorporation [19]. Additionally, soil moisture and temperature play an important role in influencing the soil processes involved in residue decomposition and nutrient cycling; thus, CC and crop residue effects on soil C and N dynamics is dependent on agroecosystem management and environmental factors.

Soil OC and total N change slowly over the long-term and are considered as the indicators of stable fraction of soil C and N, whereas SLAN (Solvita labile amino N), Solvita CO_2_-burst, microbial biomass C and N (MBC and MBN), water extractable organic C and N (WEOC and WEON), wet aggregate stability (WAS), total inorganic N, and cumulative 2d C mineralization (Cmin_2d_), are known to vary over short (seasonal) term, and thus, are the indicators of labile fraction of C and N. Seasonal variability (across the growing season) in labile indicators of C and N [20-24] is primarily dependent on the quantity of crop residue produced, rhizodeposition during crop growth, and soil temperature and precipitation, which influence the soil microbial activity and residue decomposition [25]. Microbial activity is a key factor affecting the cycling of labile pools of C [26] and N. For instance, MBC and MBN respond quickly to the addition/incorporation of crop residue in the soil. Moebius et al. [21] reported significant temporal variation in soil physical indicators, such as WAS, over a crop growing season in a tillage system. Similarly, soil respiration, water extractable fractions are very sensitive and responsive to land management practices [27,28]. Therefore, soil indicators of labile fractions of C and N are very useful for detecting the initial changes in the status of soil organic matter which affects the nutrient turnover [29]. Moreover, evaluation of short-term changes in the soil C and N indicators, especially after CCs, provides valuable information on microbial biomass dynamics, substrate availability, timing of N mineralization, and the net balance between N mineralization and immobilization. A better understanding of the seasonal dynamics of soil C and N will advance our knowledge for improving agricultural management practices focusing on enhancing agricultural sustainability, soil quality, and climate change mitigation. Thus, assessments of seasonal variability in labile indicators of soil C and N are needed.

The inclusion of CCs in crop rotations may provide greater rotational and temporal diversity in the production systems [30]; thus, resulting in an increase in microbial activity [31] cycling of C and N, and decomposition of biomass and C assimilation [32]. Furthermore, the temporal variety in the plant inputs (observed with CC in the crop rotations) and residue quantity and quality have a strong influence on soil functions and processes, which may have implications on increasing crop yields [30]. Adoption of CCs also result in an increase in the soil OC which plays an important role in enhancing the soil functionality, processes, and sustainability.

To date, there is a limited knowledge on the potential interactive effects of CCs and crop residue removal on soil C and N dynamics. Additionally, CC and residue removal impacts on soil C and N accumulation are expected to be observed in the medium- to long-term as opposed to the short-term (see reviews by [2,3,11]). Therefore, the medium-term CC experiment used in this study was ideally suited for evaluating the CC- and crop residue-induced effects on C and N dynamics. The main objective of the study was to assess the mechanism of C and N accumulation with CCs and crop residue management (removed (-R) and retained (+R)). We hypothesize that a potential synergistic effect of enhancing crop diversity by including CCs and maintaining crop residues will be observed on soil C and N sequestration. The study is expected to advance our understanding of the integrative effect of CCs and crop residue removal on soil processes involved in C and N cycling.

# **Materials and Methods**

## **Site description and experimental design**

The medium-term CC experiment, established in 2007 and repeated an adjacent site in 2008 at Ridgetown, Ontario, Canada, was used for studying soil C and N dynamics. Site-year was used to clearly indicate that soil and crop sampling was conducted from different sites in different years. Soil characteristics are described in Table 1. Since the initiation of the CC experiment in 2007 and 2008, crop rotation consisted of grain and processing vegetable crops, typical of southwestern Ontario (Table 2). As described previously [6,33-36], experimental design consisted of a split-plot arranged as a randomized complete block with four replicates. Summer-planted (July, August, September) annual CC treatments were grown in the main plots since 2007 and 2008. In addition to a no-CC, four CC treatments were evaluated which were oat (*Avena sativa* L.), oilseed radish (OSR) (*Raphanus sativus* L. var. *oleoferus* Metzg. Stokes), winter cereal rye (rye, *Secale cereale* L.), and a mixture of OSR+Rye. Cover crops were not planted after grain corn harvest in late October/early November due to the cold temperatures; after soybean harvest, winter wheat was planted instead of CCs. Crop residue management was applied in the split-plots three times since the experiment started at both sites (in 2009 and 2010 after spring wheat harvest, 2011 and 2012 after grain corn harvest, and 2014 and 2015 after winter wheat harvest) in the production system. Thus, from 2007 to 2015 and 2008 to 2016, CCs were planted six times in the crop rotation (Table 2). Cover crop attributes (biomass, C and N concentration) were quantified three times (late October/early November, April, and May) in 2014-15 and 2015-16.

**Table 1. Select soil^z^ properties from 0-15 cm depth at Ridgetown, Ontario, Canada during 2015 and 2016**

| Property^y^ | Site-year 2015 | Site-year 2016 |
| --- | --- | --- |
| Soil texture | Sandy loam (Orthic humic gleysol) | |
| Particle size distribution |  |  |
| Sand (%) | 76.7±0.26 | 76.3±0.31 |
| Silt (%) | 16.9±0.24 | 18.5±0.29 |
| Clay (%) | 6.34±0.14 | 5.07±0.20 |
| pH | 6.12±0.05 | 7.06±0.04 |
| Phosphorus (mg kg^-1^) | 5.60±0.17 | 21.4±1.55 |
| Potassium (mg kg^-1^) | 136±3.93 | 147±5.87 |

^z^ Means of a composite sample (sixty soil cores of 3.5 cm diameter) taken from each cover crop and crop residue treatment plot (n=40) at tomato harvest in September 2015 and 2016.

^y^Methods included particle size via hydrometer, pH was 1:1 (soil:water) by volume, P was Olsen bicarbonate extraction and K was ammonium acetate extraction.

**Table 2. In a medium-term cover crop experiment, crop rotation and crop residue management from 2007 to 2015 (site-year 2015) and 2008 to 2016 (site-year 2016)**

| Site-year 2015 | Site-year 2016 | Main crop | Fall planting |
| --- | --- | --- | --- |
| 2007 | 2008 | Processing pea | Cover crops |
| 2008 | 2009 | Processing sweet corn | Cover crops |
| 2009 | 2010 | Spring wheat | Cover crops |
| 2010 | 2011 | Processing tomato | Cover crops |
| 2011 | 2012 | Grain corn -stover removal | None |
| 2012 | 2013 | Processing squash | Cover crops |
| 2013 | 2014 | Soybean | Winter wheat |
| 2014 | 2015 | Winter wheat -straw removal | Cover crops |
| 2015 | 2016 | Processing tomato | Cover crops |

All the crop management and field operations have been previously described in Chahal and Van Eerd [34]. Two weeks prior to tomato transplanting, P and K fertilizers were applied at the rate of 94.1 kg ha^-1^ (P_2_O_5_) and 123 kg ha^-1^ (K_2_O) [33]. Soil was tilled on May 19, 2015 and May 20, 2016 to incorporate CC residues and fertilizers and to prepare for tomato transplanting. At tomato transplanting, a starter N fertilizer (15 kg N ha^-1^) was applied with water [6,33]. No irrigation was applied to tomato crop.

## **Soil sampling and analysis**

In both site-years, soil was sampled from 0-15 cm depth at seven different times during tomato growing season. Sampling regime was based on tillage; 3 weeks before tillage on April 29, 2015 and April 27, 2016; at tillage on May 19, 2015 and May 20, 2016; and two, four, eight and twelve weeks after tillage (WAT) on June 5, June 16, June 29, July 29 in both site-years; and at tomato harvest on September 11, 2015 and September 6, 2016. A composite sample of random 20 soil cores of 3.5 cm diameter was taken from each split-plot and soil samples were homogenized by hand in the field.

Sample handling and sieving for OC [37], total N [38], Cmin_2d_ [39], MBC and MBN [40] was similar; soil was sieved through 4 mm mesh screen and protocols described in Carter and Gregorich [41] were followed. Until processing, soil samples for OC, total N, Cmin_2d_, and total inorganic N (TIN) were stored in a cooler (4ºC); MBC and MBN were stored in the freezer (-20ºC). A sub-sample of soil (5 g for OC and total N, and 80 g for Cmin_2d_) was oven-dried at 50ºC for 24 hours. A ground (mortar and pestle) sub-sample of oven-dried soil (0.15 g) was used for quantifying OC and total N on LECO CN analyzer (Leco Corporation, St. Joseph, MI). For evaluating Cmin_2d_, substrate-induced respiration was conducted with an addition of glucose (1.6 mg C g^-1^ dry soil) using an alkali trap method with 60 g dry soil [35,42]. Soil organic C and Cmin_2d_ were expressed as mg C g^-1^ dry soil; total N and TIN were expressed as mg N g^-1^ dry soil. Microbial biomass C and N were measured by using chloroform-extraction method as described by Carter and Gregorich [41] and Vance et al. [43]. Fifteen grams of moist soil sub-sample was fumigated, in a sealed glass desiccator kept under vacuum for 24 hrs in the dark in a fume hood, using 50 mL of ethanol-free chloroform which was placed in a beaker along with boiling chips; whereas another 15 g soil was non-fumigated. Both fumigated and non-fumigated soil samples were extracted using 30 mL of 0.5 M K_2_SO_4_ solution, and filtrates were analyzed for C and N via dry combustion on a LECO CN analyzer (Leco Corporation, St. Joseph, MI). Microbial biomass C and MBN were quantified as the difference of fumigated and non-fumigated samples and were expressed as μg C g^-1^ and μg N g^-1^, respectively. The k_EC_ and k_EN_ coefficients used for MBC and MBN were 0.45 and 0.18, respectively [41,44]. Wet aggregate stability was measured by using 4 g un-sieved air-dried soil in an apparatus (Eijelkamp Agrisearch Equipment 08.13, Giesbeek, The Netherlands) similar to Kemper and Rosenau [45] and described in detail by Van Eerd et al. 2018 [46].

For Solvita CO_2_-burst, water extractable organic C (WEOC), water extractable organic N (WEON), and Solvita labile amino N (SLAN), soil was sieved through 2 mm mesh screen and 60 g soil sub-sample was oven-dried at 40ºC for 24 hrs [33,47-50]. Briefly, 4 g of oven-dried soil was used for quantifying SLAN, WEOC, and WEON; Solvita CO_2_-burst was measured using 40 g dry soil (see Chahal and Van Eerd [33] for details). For quantifying WEOC and WEON, 4 g oven-dried soil was extracted with 40 mL distilled water by mechanically shaking on an orbital shaker for 10 mins, followed by filtration and analyzing the extracts for inorganic N (on an auto analyzer SEAL Analytics) and total and inorganic C (on LECO CN analyzer) [33]. Soil with the gel paddles was incubated for 24 hrs in a sealed glass beaker to evaluate Solvita CO_2_-burst and SLAN. After 24 hr incubation, gel paddles changed the colour due to evolved CO_2_ (Solvita) and NH_3_ (SLAN); colour intensity measurements were taken using a digital colorimeter reader [33]. Concentrations of SLAN, WEON, WEOC, and Solvita CO_2_-burst were expressed on dry weight basis as mg NH_3_-N kg^-1^, mg N kg^-1^, mg C kg^-1^, and mg CO_2_-C kg^-1^.

## **Data Analysis**

All indicators were expressed on a soil dry weight concentration basis. Soil OC and total N represented the indicators of stable fractions of soil C and N, whereas all other evaluated parameters indicated the labile pools of C and N. Both site-years were evaluated separately as initial statistical analysis detected an interaction between site-year and treatments. In each site-year, fixed effects of CC, crop residue management, and sampling time, and their interactions on soil attributes were assessed using repeated measures analysis with PROC GLIMMIX in SAS (SAS Institute, version 9.4 Cary, NC, USA). Replication, and replication by CC were the random effects [51]. To account for the repeated measure analysis, a random statement of replication by sampling time was included. Analysis of residuals and normality test (Shapiro-Wilk) was conducted to confirm the assumptions of ANOVA [52]. Based on the output of studentized conditional residuals, no outliers were detected. All assumptions of ANOVA were met, therefore, no adjustments to covariance structure and data transformations were conducted. A protected LSD test was used to compare treatment means at α=0.05. Additionally, radar charts were prepared for both crop residue treatments at tomato harvest in each site-year; where data were expressed as a ratio of the average indicator value for each CC treatment and the maximum value of the respective attribute, to facilitate the visual interpretation of the impact of management on soil parameters. As mentioned previously, microbial activity is a critical function governing soil organic matter formation [30]; therefore, Pearson correlations of OC-related processes (MBC, MBN, and WAS) with labile indicators (Cmin_2d_, WEOC, WEON, TIN, Solvita CO_2_-burst and SLAN) stable indicators (OC, total N) were assessed.

## **Tomato growing season**

Air temperature and precipitation during the tomato growing season in site-years 2015 and 2016 were collected by an Environment Canada weather station located < 1 km from the experiment (Fig 1). Spring 2016 (April and May) was drier than spring 2015 by 69 mm (Fig 1) and 30-yr mean by 52.8%, whereas spring 2015 had a relatively similar total precipitation (74.7 mm) as 30-yr mean (76 mm). Average monthly air temperature during most of the tomato growing season from June to August was comparable in both site-years (mean 24.7 ºC in site-year 2015; mean 25.8 ºC in site-year 2016) but warmer than 30-yr mean (21.4 ºC). In contrast to air temperature, total monthly precipitation from June to August in sampling years was lower than 30-yr mean by 20.3 and 19.3 mm, respectively. However, total precipitation during tomato growing season in both years was equivalent (65.7 mm in 2015 and 65.5 mm in 2016; Fig 1). Regarding the temporal distribution of precipitation relative to soil sampling, June and July had the greatest total precipitation in site-year 2015, while early July was greatest in site-year 2016 (Fig 1). Despite differences in weather between years, in both site-years there were no effects of CC and crop residue management treatment on soil gravimetric moisture content (S1 Table). In site-year 2015, soil moisture content in April was greater than June, July, and September. No seasonal variability in soil gravimetric moisture content was detected in site-year 2016.

**Fig 1.** **Daily total precipitation (mm) and mean air temperature (ºC) during the tomato growing season in 2015 and 2016 at Ridgetown, Ontario, Canada. Dash lines with arrows represent the soil sampling dates in site-year 2015 and 2016.**

# **Results**

## **Cover crop attributes**

In fall of both site-years, OSR had the greatest CC biomass, C and N content (avg. 4850 kg ha^-1^, 1780 kg C ha^-1^, 159 kg N ha^-1^, respectively) whereas cereal rye had the lowest (1960 kg ha^-1^, 824 kg C ha^-1^, and 64 kg N ha^-1^ , respectively; Fig 2). Over 6-yrs from fall 2007 to fall 2014, average annual CC biomass across all CC treatments was 3220±115 kg ha^-1^, C was 1280±46.2 kg C ha^-1^, and N was 78±3.47 kg N ha^-1^ (Fig 2). Similarly, from fall 2008 to fall 2015, average annual CC biomass across all CC treatments was 3520±116 kg ha^-1^, C was 1290±50.7 kg C ha^-1^, and N was 97.7±4.15 kg N ha^-1^ (Fig 2). Cover crop C concentration was not different among CC treatments in both springs (2015 and 2016, S2 Table). In spring 2015, C:N of CC was 18.4-32.3 with the trend of OSR≥OSR+Rye=rye≥oat (S2 Table). In contrast, C:N of CC in spring (avg. 19.2±1.54) in site-year 2016 was not different among CC treatments (S2 Table).

**Fig 2. In a medium-term cover crop (CC) experiment, annual above-ground CC biomass, C and N inputs in fall 2014, fall 2015, and average over a period of 6-yrs from various CC treatments. For each parameter in each year, bars followed by a different letter indicate statistical significance per protected LSD test (*P*<0.05; n=8).**

**Soil attributes**

In both site-years, concentration of total N did not change over the tomato growing season (Tables 3 and 4). However, a significant CC by time interaction for OC was detected in both site-years (Tables 3 and 4) which was attributed to the lowest OC concentration in no-CC plots at tomato harvest. In both site-years, CC treatment differences, but no two-way interaction of CC by time, were detected on total N concentration with no-CC being the lowest (S3 and S4 Tables). In addition to medium-term CC effects on OC and total N, the +R treatment had greater OC concentration than -R in site-year 2015 (*P* < 0.0001) but no effect in site-year 2016 (*P* = 0.9888).

**Table 3. Probability values for the main effects of medium-term summer-planted cover crop (6-yrs), crop residue management (3-yrs), sampling time, and interactions on soil indicators sampled from 0-15 cm depth in site-year 2015**

|  |  | Cover crop (CC) | Crop residue (R) | Time (T) | CC x R | CC x T | R x T | CC x R x T |
| --- | --- | --- | --- | --- | --- | --- | --- | --- |
| Indicator | Unit | -------------------------------------------------Pr > F ------------------------------------------- | | | | | | |
| OC | mg C g^-1^ | **<0.0001** | 0.0538 | 0.1969 | 0.1708 | **<0.0001** | **<0.0001** | 0.0903 |
| Total N | mg N g^-1^ | **<0.0001** | **<0.0001** | 0.1337 | 0.0782 | 0.2797 | 0.4344 | 0.8157 |
| WAS | % | **<0.0001** | **<0.0001** | **<0.0001** | **<0.0001** | **<0.0001** | 0.3619 | **0.0126** |
| Cmin_2d_ | mg C g^-1^ | **<0.0001** | **<0.0001** | **<0.0001** | 0.5802 | **<0.0001** | **0.0002** | 0.9904 |
| Solvita | mg CO_2_-C kg^-1^ | **0.0138** | **0.0011** | **<0.0001** | 0.6759 | **<0.0001** | 0.7229 | 0.9986 |
| SLAN | mg NH_3_-N kg^-1^ | **<0.0001** | 0.7781 | **<0.0001** | **0.0038** | **<0.0001** | **<0.0001** | **0.0460** |
| WEOC | mg C kg^-1^ | **<0.0001** | **0.0027** | 0.6753 | **<0.0001** | **0.0037** | 0.0720 | **0.0015** |
| WEON | mg N kg^-1^ | 0.1496 | 0.0587 | 0.1765 | 0.0511 | **0.0189** | 0.1469 | 0.1316 |
| Total inorganic N | mg N g^-1^ | **<0.0001** | **0.0048** | 0.2873 | **<0.0001** | **0.0059** | 0.2318 | **0.0027** |
| MBC | μg C g^-1^ | **<0.0001** | 0.7000 | **<0.0001** | 0.7589 | **0.0159** | 0.7386 | 0.9591 |
| MBN | μg N g^-1^ | **<0.0001** | 0.7830 | **<0.0001** | 0.9213 | **0.0302** | 0.7369 | 0.9205 |

Bold font indicates statistical significance at *P*<0.05

Cmin_2d_, cumulative 2d soil carbon mineralization; MBC, microbial biomass C; MBN, microbial biomass N; SLAN, Solvita labile amino N; OC, soil organic C; WAS, wet aggregate stability; WEOC, water extractable organic C; WEON, water extractable organic N.

**Table 4. Probability values for the main effects of medium-term summer-planted cover crop (6-yrs), crop residue management (3-yrs), sampling time, and interactions on soil indicators sampled from 0-15 cm depth in site-year 2016**

|  |  | Cover crop (CC) | Crop residue (R) | Time (T) | CC x R | CC x T | R x T | CC x R x T |
| --- | --- | --- | --- | --- | --- | --- | --- | --- |
| Indicator | Unit | -----------------------------------------------------Pr > F ------------------------------------------------- | | | | | | |
| OC | mg C g^-1^ | **0.0003** | 0.8278 | 0.1055 | 0.4242 | **<0.0001** | 0.9888 | 0.7518 |
| Total N | mg N g^-1^ | **<0.0001** | 0.7065 | 0.0536 | 0.5112 | 0.4459 | 0.4296 | 0.4355 |
| WAS | % | **0.0122** | **<0.0001** | **0.0002** | 0.7240 | **<0.0001** | 0.6911 | 0.9501 |
| Cmin_2d_ | mg C g^-1^ | 0.6454 | 0.2510 | **<0.0001** | 0.0581 | 0.1546 | 0.8764 | 0.9965 |
| Solvita | mg CO_2_-C kg^-1^ | **<0.0001** | 0.6009 | **<0.0001** | 0.7403 | **<0.0001** | 0.0985 | 0.8098 |
| SLAN | mg NH_3_-N kg^-1^ | **<0.0001** | 0.5351 | **<0.0001** | **<0.0001** | **<0.0001** | **0.0119** | **<0.0001** |
| WEOC | mg C kg^-1^ | **<0.0001** | **<0.0001** | **<0.0001** | **<0.0001** | **0.0009** | 0.2326 | 0.4115 |
| WEON | mg N kg^-1^ | 0.0708 | 0.2796 | **<0.0001** | 0.1069 | 0.7595 | 0.4980 | 0.3860 |
| Total inorganic N | mg N g^-1^ | **0.0001** | **<0.0001** | **<0.0001** | **<0.0001** | **0.0254** | **<0.0001** | 0.8429 |
| MBC | μg C g^-1^ | **<0.0001** | 0.8096 | 0.1674 | 0.5194 | **0.0254** | 0.6503 | 0.6044 |
| MBN | μg N g^-1^ | **<0.0001** | 0.2688 | 0.0892 | 0.1489 | 0.2379 | 0.5648 | 0.1883 |

Bold font indicates statistical significance at *P*<0.05

Cmin_2d_, cumulative 2d soil carbon mineralization; MBC, microbial biomass C; MBN, microbial biomass N; SLAN, Solvita labile amino N; OC, soil organic C; WAS, wet aggregate stability; WEOC, water extractable organic C; WEON, water extractable organic N.

In contrast to stable indicators, labile C and N indicators varied temporally with CC and crop residue management. Seasonal variability with CCs was detected in all labile indicators in site-year 2015 (Table 3), but in site-year 2016, 6 out of 9 indicators changed temporally with CCs (Table 3). Moreover, compared with the CCs, interaction between crop residue management and time was detected in only two labile indicators (SLAN and Cmin_2d_ in site-year 2015, SLAN and total inorganic N in site-year 2016; Tables 3 and 4). Across tomato growing season, no-CC had the lowest Cmin_2d_, MBC, and MBN concentrations in site-year 2015 (Fig 3), and the lowest MBC and Solvita CO_2_-burst concentration in site-year 2016 (Fig 4). The CC by time interaction in site-year 2015 for Solvita CO_2_-burst, MBC, MBN, and Cmin_2d_ was due to lowest concentrations in no-CC (oat intermediate for Solvita, Cmin_2d_) in April but these differences were minimized at other time points (Fig 3). A sharp decrease was observed in the no-CC treatment only for MBC concentration from April to 2WAT followed by a levelling in the values until tomato harvest in site-year 2016 (Fig 4). For WAS, greater CC treatment differences were detected at April in site-year 2016 (Fig 4). As expected, WEON was highly variable in its temporal response to CCs and hence, no clear trend was detected in CC treatments across different sampling times (Fig 3). Greater concentration of labile indicators with CC than no-CC across the season confirm the positive influences of CC on C and N dynamics in our medium-term experiment.

**Fig 3. In site-year 2015, effect of medium-term summer-planted cover crop (6-yrs) on soil indicators from 0-15 cm depth over the tomato growing season. Bars represent standard error of means (n=8)**

Cmin_2d_, cumulative 2d soil C mineralization; MBC, microbial biomass C; MBN, microbial biomass N; WEON, water extractable organic N

**Fig 4. Over the tomato growing season, effect of medium-term summer-planted cover crop (6-yrs) on soil indicators from 0-15 cm depth in site-year 2016. Bars represent standard error of means (n=8).**

MBC, microbial biomass C; WAS, wet aggregate stability; WEOC, water extractable organic C

Retaining crop residues in the field resulted in greater concentrations of WAS, Cmin_2d_, Solvita CO_2_-burst, and total N in site-year 2015, and WAS, total inorganic N, and WEOC in site-year 2016 than removing residues (S3 and S4 Tables). Over the season, +R had greater concentrations of labile indicators (Cmin_2d_ in site-year 2015 and total inorganic N in 2016) than -R. For Cmin_2d_, interaction between crop residue treatments and time was due to greater values in +R than -R at all sampling times, except tomato harvest. In contrast, total inorganic N concentration in April was not different between +R and -R, while +R had greater concentration than -R across all other sampling times; thus, leading to an interaction between sampling time and crop residue treatments (data not shown). Therefore, these results of greater concentrations of indicators with +R than -R further indicates the potential of +R in increasing soil fertility, functionality, and C and N cycling.

In addition to the two-way interactions described above, three-way interactions between CC, crop residue treatment, and sampling time (Table 3 and 4) were detected on SLAN in both site-years, and WAS, WEOC, and TIN in site-year 2015 (Fig 5). In both site-years, no-CC+R and no-CC-R had the greatest SLAN concentration at all time points (Fig 5). In site-year 2015 only, the least differences in SLAN concentrations among treatments were observed at harvest (Fig 5). Least treatment differences for WEOC and total inorganic N were detected in mid-June to July (Fig 5). As expected, WEOC and total inorganic N were highly variable temporally; no clear trend was detected in CC and crop removal treatments, however, cereal rye had the least concentration over the season (Fig 5). Therefore, these results of variable response of soil attributes due to CC and crop residue treatments over the growing season confirms the dynamic nature of soil C and N indicators.

**Fig 5. In site-year 2015 (A-D) and site-year 2016 (E), effect of medium-term summer-planted cover crop (6-yrs) and crop residue retained (+R) or removed (-R) (3-yrs) on soil indicators from 0-15 cm depth over the tomato growing season. Bars represent standard error of means (n=4)**

SLAN, Solvita labile amino N; WAS, wet aggregate stability; WEOC, water extractable organic C; WEON, water extractable organic N

To facilitate comparison of CC and crop residue management on soil C and N indicators, radar charts were prepared for each site-year at tomato harvest (i.e., early September, Fig 6). In both site-years, even at one time-point (at tomato harvest representing cumulative effect of CC and crop residue treatments), differences in stable and labile indicators of C and N were detected with CC and crop residue treatments. Overall, in both site-years, the -R treatment had fewer differences among CC treatments than +R. No cover crop control had the lowest values of MBC, MBN, total N, OC, WAS, and Cmin_2d_ in both crop residue treatments in site-year 2015 (Fig 6). In addition to aforementioned indicators, no-CC was lowest for Solvita CO_2_-burst in +R and -R in site-year 2016 (Fig 6). Overall between both site-years and crop residue treatments, cereal rye had the greatest values of Cmin_2d_, MBC, MBN, OC, total N, and Solvita CO_2_-burst. These CC effects (i.e, no-CC lowest MBC, MBN, total N, and OC but greatest SLAN) and crop residue effects on soil attributes at tomato harvest were consistent with treatment effects observed temporally over the crop growing season.

**Fig 6. Effect of medium-term summer-planted cover crop (6-yrs) and crop residue retained (+R) (A,C) or removed (-R) (B,D) on soil indicators at tomato harvest in site-year 2015 (A,B) and 2016 (C,D). Values in parenthesis represent mean (n=4) maximum indicator concentrations expressed as mg g^-1^ for Cmin_2d_, OC, TIN, and TN; mg kg^-1^ for WEOC, WEON, Solvita, and SLAN; μg g^-1^ for MBC and MBN; % for WAS (Cmin_2d_, cumulative 2d soil C mineralization; MBC, microbial biomass C; MBN, microbial biomass N; SLAN, Solvita labile amino N; OC, organic C; TIN, total inorganic N; TN, total N; WAS, wet aggregate stability; WEOC, water extractable organic C; WEON, water extractable organic N).**

Across all the sampling times in site-year 2015 and 2016, correlation analysis was conducted between soil indicators (S5 and S6 Tables). In site-year 2015, MBC correlated negatively with Solvita CO_2_-burst (*P* < 0.0001, r=-0.37), SLAN (*P* <0.001, r=-0.34), total inorganic N (*P* =0.0004; r=-0.21), and WEOC (*P* = 0.0027; r=-0.17) and positively with Cmin_2d_ (*P* < 0.0001; r=0.31). Similarly, in site-year 2015, MBN correlated negatively with Solvita CO_2_-burst (*P* < 0.0001, r=-0.32), SLAN (*P* <0.001, r=-0.35), total inorganic N (*P* =0.0016; r=-0.18), and WEOC (*P* = 0.0092; r=-0.15) and positively with Cmin_2d_ (*P* < 0.0001; r=0.32).

Contrary to site-year 2015, in site-year 2016 there were fewer significant correlations (S6 Table). The only significant positive correlations were between WAS and Cmin_2d_ (*P* = 0.0189, r=0.14), MBC and Solvita CO_2_-burst (*P* < 0.0001, r= 0.57), MBN and Cmin_2d_ (*P* = 0.0208, r= 0.13), and MBN and Solvita CO_2_-burst (*P* < 0.0001, r=0.41). Moreover, correlations between OC-related processes (MBC, MBN, and WAS) and stable indicators (OC, total N) were assessed for both site-years (S5 and S6 Tables). Organic C correlated positively with WAS (*P* < 0.0001, r=0.24) in site-year 2015 but not in site-year 2016, whereas MBC (*P* ≤ 0.0029, r= 0.17 to 0.57) and MBN (*P* ≤ 0.0028, r=0.17 to 0.41) correlated positively with total N in both site-years (S5 and S6 Tables). These correlations (positive or negative) between the short- and long-term indicators of management further confirm the close associations between the labile and stable attributes of C and N and their processes in our medium-term CC experiment.

# **Discussion**

In spring 2015 and 2016, CC C:N ratios were favourable for mineralization for all CCs (18.4 to 24.8) but OSR (32.3) in spring 2015 only. Similar C:N of CC was previously reported in this CC experiment[6,35] and elsewhere [11]. Our results of greatest CC biomass from OSR in fall 2014 and 2015 and OSR+Rye in spring 2015 and 2016 were consistent with the previous studies in the same medium-term CC experiment [6,34,35]. In this medium-term experiment, annual CC biomass production and C and N contents were different at each fall sampling (from fall 2007 to fall 2015 and from fall 2008 to fall 2016). This difference in fall CC biomass (and C and N content) over the study duration was due to timing of main crop harvested (i.e. fresh processing pea in late July and processing tomato in early Sept) and consequently the length of the CC growing season.

Cover crops are known to increase the concentrations of stable pools of C and N in the long-term [53] but this effect is not observed in the short-term [54]. Moreover, several studies have demonstrated the strong influence of CC C and N inputs over the medium- and long-term in increasing the stable pools of soil C and N [53,55,56]. In our study, stable indicators of soil C and N (OC and total N) were influenced by CC and crop residue management but did not change temporally. We observed that, across all sampling times, incorporation of CC C and N inputs significantly increased the soil OC and total N concentration compared with the no-CC. A similar effect of CC treatments on soil OC and total N was reported by Zhou et al [57]. Likewise, Sainju et al. [53,56] observed least total N concentration from no-CC. Lowest concentration of total N and OC in no-CC treatment could be attributed to lesser C and N biomass inputs than CCs in the medium-term. In our medium-term experiment, average annual CC above-ground C inputs were 1280 to 1380 kg C ha^-1^ and CC above-ground N inputs were 78 to 97 kg N ha^-1^, which contributed to a significant increase in soil OC and total N. Our observed greater OC and total N concentration with CCs and crop residue retention indicates the potential of these management practices to increase stable indicators of soil C and N in the medium-term and beyond.

As expected, indicators of labile fractions of soil C and N (WAS, WEOC, WEON, MBC, MBN, SLAN, Cmin_2d_, Solvita CO_2_-burst, and total organic N) changed with CC and crop residue treatments over the tomato growing season indicating the dynamic nature of these indicators. Cover crop residue contributes to the labile soil organic matter, thus, affecting the C and N dynamics [58]. Labile pools of soil C and N are crucial for soil biology as they act as a nutrient reservoir for soil microbes [57]. Seasonal variation in the labile indicators observed might be attributed to the root exudates released from the tomato roots. Labile C compounds released by the roots, rhizodepositions, are reported to be greater at flowering than planting stage, are incorporated into the microbial biomass; thus, directly impact the microbial community structure and functionality and enhance the concentrations of labile pools of C and N. Several studies have reported that root C and N inputs are largely responsible for influencing the soil biological activity [59,60]. As the crop matures, the concentration of rhizo-deposits decreases but fresh crop residues and mature roots are added to the soil which contribute to the microbial substrates, therefore, increasing the labile fractions of C and N at harvest [61]. Overall, our observed variation in the concentrations of labile indicators across the growing season and between years might be attributed to the differences in the quantity of CC biomass produced among the tested CCs and the interactions between soil microbes and plant roots. Lack of a clear distinction among the tested CC species in impacting the soil indicators and processes, such as respiration, across the season confirm the dominance of CC biomass (C inputs) in controlling the soil C and N dynamics than the quality (CC C:N) in this medium-term experiment.

Moreover, weather differences (air temperature and precipitation) between sampling years might have resulted in the temporal variation in the labile indicators of soil C and N. Similarly, seasonal differences in the labile indicators in response to variable frequency and intensity of precipitation were reported by Hui et al. [62]. Site-year 2016 was warmer in spring (April to May) and drier (from April to June) than 2015 (Fig 1), which likely impacted the microbial activity, residue decomposition, nutrient uptake, availability, and release. Generally, a direct and positive relationship is observed between temperature and microbial activity [63], while soil moisture content in the range of 50-70% of water holding capacity is preferred for microbial functions and processes [64]. Greater microbial activity results in an increase in the potential of residue decomposition. Our result of high MBC and MBN in early spring, perhaps due to low soil temperature and high soil moisture, was consistent with several studies [22-24]. Additionally, June 2015 had higher total precipitation than June 2016, which might have impacted the microbial mediated soil functions and processes between both site-years. Overall, high precipitation in July of both site-years was beneficial for the tomato crop as it matched with the period of greatest water demand by the crop.

Out of all the sampling times evaluated in this study, 2WAT (early June) and harvest (early September) is recommended for sampling soil C and N indicators. In one out of two site-years, 2WAT had greater concentrations of Solvita CO_2_-burst, WEON, Cmin_2d_, and SLAN for all CC treatments than other sampling times, which might be attributed to an increase in the microbial activity and nutrient cycling due to residue incorporation. Tillage increases soil aeration and temperature, breaks soil macro and micro-aggregates; thus, exposes the physically protected soil OC [65] and increases the availability of OC to microbes. These processes result in increasing microbial activity and labile C and N parameters [66]. In agreement with our results, several studies [67-69] have reported that labile fractions of C and N have greater sensitivity to tillage than soil OC and total N; hence, have implications to be used as soil quality indicators. Moreover, early June (2WAT) represented the medium- (CC) and short- (residue incorporation) term effects on the concentrations of soil indicators. Similar increases in the indicator concentrations following residue incorporation has been reported by Sainju et al. [7] in a CC based tomato production system. In contrast to June, microbial activity would be expected to be slower in April due to less availability of substrate and cool soil temperature, leading to the observed lower concentrations of soil indicators. Hence, April may not be an optimum time for soil C and N sampling. Additionally, compared with other sampling times, greater treatment differences in soil indicator concentrations (WEOC, total inorganic N, Solvita CO_2_-burst, SLAN, WAS, and MBC) were detected at tomato harvest. Greater concentration of soil indicators with CC than no-CC at tomato harvest might be a reflection of a build up of stable and labile pools of C and N due to the cumulative effects of 6-yrs of CCing. Therefore, June and/or September in a crop growing season is a reasonable time for soil sampling to evaluate soil C and N dynamics and overall soil quality.

Compared to other CC treatments, lowest values of WAS, MBC, MBN, Solvita CO_2_-burst, and Cmin_2d_ were observed with no-CC (S3 and S4 Tables). A review by Blanco-Canqui et al. [11] reported greater percentage of water stable aggregates with CC than without. Soil aggregation is known to be impacted by the CC and crop residue retention due to an increased production of organic binding agents resulting in stabilization of aggregates [11,70]. Plant roots result in the formation of stable aggregates via the mechanisms of (a) increased production of polysachharides, (b) trapping of soil particles between the root hairs, and (c) increased concentration of chemicals responsible for stabilizing micro and macroaggregates [71-73]. Similarly, several studies have reported the increases in MBC, MBN, and soil respiration (evolved CO_2_) with CCs [74-77].

In only one of two site-years, -R lowered Cmin_2d_, Solvita CO_2_-burst, total inorganic N, and WEOC, which could be attributed to a reduction in microbial activity and C inputs with the crop residue removal [2]. Likewise, WAS was consistently lowest from -R plots in both site-years, which concurs with other research [5,13,70]. Inclusion of CC in cropping systems has shown to offset the negative effects of residue removal on soil indicators [78]; however, the extent to which CC mitigate the residue removal effects were highly dependent on CC biomass produced, duration of study, soil texture, initial OC concentration, and climatic conditions [2]. In contrast to removal, crop residue (winter wheat) retention (high C:N (80)) likely result in N immobilization ,reduced crop N uptake, and decrease crop yield. But, the lack of any negative impacts of crop residue management on crop yield and C and N dynamics in our study might be attributed to inherently rich and fertile soil at our experimental sites.

Due to less differences observed in the CC parameters (quantity and quality of aboveground CC biomass) in our study, another mechanism (other than inputs) might be influencing the soil C and N pools and processes. Dignac et al. [79] also indicated the possibility of another abiotic OC stabilization mechanisms such as protection of OC within the minerals, adsorption and interaction of OC with the minerals, and physical protection of OC within the aggregates. Physical mechanisms of soil OC protect the degradation of OC by the microbes and help in the stabilization of soil OC in the long-term [80]. It is not clear if the observed increases in indicator concentrations with CC and crop residue retention were due to minimizing C and N losses, protecting soil OC in micro-aggregates, preventing decomposition of organic matter, and/or influencing rhizodepositions and microbial community structures. Moreover, belowground biomass is another major contributor to soil OC concentrations, which was not measured in our study. Thus, future research elucidating the mechanism of CC (above- and below- ground biomass and root exudates) and crop residue effects on soil C and N cycling and storage is needed.

Additionally, the effects of land management practices on the mechanisms of soil C and N is not fully understood. Several meta-analyses and long-term studies have shown that the intensity of the mechanisms and processes affecting the dynamics and stabilization of soil C and N changes with time, land use, and climatic conditions [16,79-82]. Moreover, the processes governing the cycling of soil C and N are affected by the interactions with other nutrients present in the soil organic matter such as phosphorus, sulphur etc. [79]. Therefore, the aforementioned factors (temporal and environmental) should be considered while studying the processes and mechanisms influencing the soil C and N stabilization.

# **Conclusions**

Soil C and N indicators reflected both short- and medium-term dynamics in our study. Temporal variations in soil parameters were attributed to the short-term impact of CCs and crop residues on labile indicators of soil C and N (MBC, MBN, SLAN, Solvita CO_2_-burst, WEOC, WEON, total inorganic N, and Cmin_2d_). The cumulative medium-term CC effect was greater concentrations of stable pools of soil C and N (OC and total N) with CC than without CC. Our observed differences (within and between site-years) in indicator concentrations with CC and crop residue treatments might not be exclusive to the quantity and quality of CC biomass produced in this study, suggesting the possibility of another mechanism influencing soil C and N dynamics. Thus, future research evaluating the mechanism governing the pathway of soil C and N losses and gains with CC and crop residue management is needed.

Overall, compared to no-CC and crop residue removal treatments, CCs and crop residue retention had greater concentrations of soil parameters temporally indicating the impact of soil C inputs on microbial processes with implications on soil quality. This study improves our understanding of CC and crop residue removal effects on stable and labile indicators of C and N and indicates the potential of CC to offset partially the potential negative impacts of crop residue removal on soil quality in the medium-term. Additionally, our results demonstrated the role of CCs in increasing OC and total N concentration after 6-yrs of CCing, and highlighted the potential of CCs in sequestering soil C and N with implications on mitigating climate change. Future research focusing on CC effects after crop residue removal with diverse land management practices, soil types, and cropping systems would enhance our understanding of the C and N cycling.

# **Acknowledgements**

Authors are grateful to Ontario Ministry of Agriculture, Food, and Rural Affairs (OMAFRA), Grain Farmers of Ontario (GFO), and Ontario Processing Vegetable Growers (OPVG) for providing research funding to LVE. We appreciate the assistance of research technician, Mike Zink and co-op students for helping in the field and lab analysis.

# **Author contributions:**

Original draft preparation: Inderjot Chahal

Review, revising, and editing: Inderjot Chahal and Laura L. Van Eerd

# **References**

1 Moore EB, Wiedenhoeft MH, Kaspar TC, Cambardella CA. Rye Cover Crop Effects on Soil Quality in No-Till Corn Silage–Soybean Cropping Systems. Soil Sci Soc Am J. 2014; 78: 968–976.

2 Ruis SJ, Blanco-Canqui H. Cover crops could offset crop residue removal effects on soil carbon and other properties: A review. Agron J. 2017; 109: 1785-1805.

3 Blanco-Canqui H, Lal R. Crop residue removal impacts on soil productivity and environmental quality. Crit Rev Plant Sci. 2009; 28: 139-163.

4 Wang J, Sainju UM. Soil carbon and nitrogen fractions and crop yields affected by residue placement and crop types. PLoS ONE. 2014; 9: e105039.

5 Wegner BR, Kumar S, Osborne SL, Schumacher TE, Vahyala IE, Eynard A. Soil response to corn residue removal and cover crops in Eastern South Dakota. Soil Sci Soc Am J. 2015; 79: 1179– 1187.

6 Belfry KD, Trueman C, Vyn RJ, Loewen SA, Van Eerd LL. Winter cover crops on processing tomato yield, quality, pest pressure, nitrogen availability, and profit margins. PLoS ONE. 2017; 12: e0180500.

7 Sainju UM, Singh BP, Whitehead WF. Cover crops and nitrogen fertilization effects on soil carbon and nitrogen and tomato yield. Can J Soil Sci. 2000; 80: 523–532.

8 Sainju UM, Singh BP, Whitehead WF. Comparison of the effects of cover crops and nitrogen fertilization on tomato yield, root growth, and soil properties. Sci Hort. 2001; 91: 201-214.

9 Summers CF, Park S, Dunn AR, Rong X, Everts KL, Meyer SLF, et al. Single season effects of mixed species cover crops on tomato health (cultivar Celebrity) in multi-state field trials. Appl Soil Ecol. 2014; 77: 51-58.

10 Blanco-Canqui H, Holman JD, Schlegel AJ, Tatarko J, Shaver T Replacing fallow with cover crops in a semiarid soil: Effects on soil properties. Soil Sci Soc Am J. 2013; 77: 1026–1034.

11 Blanco-Canqui H, Shaver TM, Lindquist JL, Shapiro CA, Elmore RW, Francis CA, et al. Cover crops and ecosystem services: Insights from studies in temperate soils. Agron J. 2015; 107: 2449-2474.

12 Steele MK, Coale FJ, Hill RL. Winter annual cover crop impacts on no-till soil physical properties and organic matter. Soil Sci Soc Am J. 2012; 76: 2164–2173.

13 Coppens F, Merckx R, Recous S. Impact of crop residue location on carbon and nitrogen distribution in soil and in water-stable aggregates. Eur J Soil Sci. 2006; 57: 570–582.

14 Ding G, Liu X, Herbert S, Novak J, Amarasiriwardena D, Xing B Effect of cover crop management on soil organic matter. Geoderma. 2006; 130: 229–239.

15 Ghimire B, Ghimire R, VanLeeuwen D, Mesbah A. Cover crop residue amount and quality effects on soil organic carbon mineralization. Sustainability. 2017; 9: 2316; doi:10.3390/su9122316

16 Drinkwater LE, Snapp S. Nutrients in agroecosystems: rethinking the management paradigm. Adv Agron. 2007; 92: 163-186.

17 Ranells NA, Wagger MG. Nitrogen release from grass and legume cover crop monocultures and bicultures. Agron J. 1996; 88: 777-782.

18 Wagger MG. Time of desiccation effects on plant composition and subsequent nitrogen release from several winter annual cover crops. Agron J. 1989; 81: 236–241.

19 Smith MS, Frye WW, Varco JJ. Legume winter cover crops. Adv Soil Sci. 1987; 7: 95-139.

20 Jiang PK, Xu QF, Xu ZH, Cao ZH. Seasonal changes in soil labile organic carbon pools within a phyllostachys praecox stand under high rate fertilization and winter mulch in subtropical China. For Ecol Manage. 2006; 236: 30–36.

21 Moebius BN, van Es HM, Schindelbeck RR, Idowu OJ, Clune DJ, Thies JE. Evaluation of laboratory-measured soil properties as indicators of soil physical quality. Soil Sci. 2007; 172: 895–912.

22 Omer M, Idowu OJ, Ulery AL, VanLeeuwen D, Guldan SJ. Seasonal changes of soil quality indicators in selected arid cropping systems. Agric. 2018; 8: 124; doi:10.3390/agriculture8080124.

23 Piao HC, Hong YT, Yuan ZY. Seasonal changes of microbial biomass carbon related to climatic factors in soils from karst areas of southwest China. Biol Fertil Soils. 2000; 30: 294–297.

24 Ryan J, Masri S, Singh M. Seasonal changes in soil organic matter and biomass and labile forms of carbon as influenced by crop rotations. Commun Soil Sci Plant Anal. 2009; 40: 188–199.

25 Xiang S, Doyle A, Holden PA, Schimel JP. Drying and rewetting effects on C and N mineralization and microbial activity in surface and subsurface California grassland soils. Soil Biol Biochem. 2008; 40: 2281–2289.

26 Christ MJ, David MB. Temperature and moisture effects on the population of dissolved organic carbon in a spodosol. Soil Biol Biogeochem. 1996; 28: 1191-1199.

27 Huang ZQ, Xu ZH, Chen CR. Effect of mulching on labile soil organic matter pools, microbial community functional diversity and nitrogen transformations in two hardwood plantations of subtropical Australia. Appl Soil Ecol. 2008; 40: 229–239.

28 Rui YC, Wang SP, Xu ZH, Wang YF, Chen CR, Zhou XQ, et al. Warming and grazing affect soil labile carbon and nitrogen pools differently in an alpine meadow of the Qinghai-Tibet Plateau in China. J Soils Sediments. 2011; 11: 903–914.

29 Salinas-Garcia JR, Hons FM, Matocha JE, Zuberer DA. Soil carbon and nitrogen dynamics as affected by long-term tillage and nitrogen fertilization. Biol Fert Soils. 1997; 25: 182-188.

30 McDaniel MD, Grandy AS, Tiemann LK, Weintraub MN. Crop rotation complexity regulates the decomposition of high- and low-quality residues. Soil Biol Biogeochem. 2014; 78: 243-254.

31 Zak DR, Holmes WE, White DC, Peacock AD, Tilman D. Plant diversity, soil microbial communities, and ecosystem function: are there any links? Ecol. 2003; 84: 2042-2050.

32 Drinkwater LE, Schipanski M, Snapp S, Jackson LE. Ecologically based nutrient management. Agric Sys. 2017; p. 203-257. <https://doi.org/10.1016/B978-0-12-802070-8.00007-4>

33 Chahal I, Van Eerd LL. Evaluation of commercial soil health tests using a medium-term cover crop experiment in a humid, temperate climate. Plant Soil. 2018; <https://doi.org/10.1007/s11104-018-3653-2>

34 O’Reilly KA, Lauzon JD, Vyn RJ, Van Eerd LL. Nitrogen cycling, profit margins and sweet corn yield under fall cover crop systems. Can J Soil Sci. 2012; 92: 353-365.

35 Ouellette L, Voroney RP, Van Eerd LL. DRIFT spectroscopy to assess cover crop and corn stover decomposition in lab-incubated soil. Soil Sci Soc Am J. 2016; 80: 284-293.

36 Chahal I, Van Eerd LL. Quantifying soil quality in a horticultural-cover cropping system. Geoderma. 2019; 352: 38-48.

37 Skjemstad JO, Baldock JA. Total and organic carbon. In M.R. Carter, and E.G. Gregorich (eds.) Soil Sampling and Methods of Analysis. CRC Press. Boca Raton, FL; 2008. p. 225-238.

38 Rutherford PM, McGill WB, Arocena JM, Figueiredo CT. Total nitrogen. In M.R. Carter, and E.G. Gregorich (eds.) Soil Sampling and Methods of Analysis. CRC Press. Boca Raton, FL; 2008. p. 239-250.

39 Hopkins DW. Carbon mineralization. In M.R. Carter, and E.G. Gregorich (eds.) Soil Sampling and Methods of Analysis. CRC Press. Boca Raton, FL; 2008. p. 589-590.

40 Voroney RP, Brookes PC, Beyaert RP. Soil microbial biomass C, N, P, and S. In M.R. Carter, and E.G. Gregorich (eds.) Soil Sampling and Methods of Analysis. CRC Press. Boca Raton, FL; 2008. p. 637-656.

41 Carter MR, Gregorich EG. Measuring change in soil organic carbon storage. In M.R. Carter, and E.G. Gregorich (eds.) Soil Sampling and Methods of Analysis. CRC Press. Boca Raton, FL; 2008.

42 Congreves KA, Voroney RP, O’Halloran IP, Van Eerd LL. Broccoli residue-derived nitrogen immobilization following amendments of organic carbon: an incubation study. Can J Soil Sci. 2013; 93: 23-31.

43 Vance ED, Brookes PC, Jenkinson DS. An extraction method for measuring soil microbial biomass C. Soil Biol Biochem.1987; 19: 703-707.

44 Joergensen RG. The fumigation-extraction method to estimate soil microbial biomass: calibration of the k_EC_ value. Soil Biol Biochem. 1996; 28: 25-31.

45 Kemper WD, Rosenau RC. Aggregate stability and size distribution. Pg. 425-442 *in* A. Klute eds. Methods of soil analysis. Part 1. 2^nd^ ed. Agronomy Monograph. 9. ASA and SSSA; 1986. Madison, WI.

46 Van Eerd LL, DeBruyn AH, Ouellette L, Hooker DC, Robinson DE. Quantitative and qualitative comparison of three wet aggregate stability methods using a long-term tillage system and crop rotation experiment. Short communication. Can J Soil Sci. 2018; 98:738-742.

47 Brinton W. Official instructions for Solvita SLAN (Solvita Labile Amino Nitrogen – lab method). Version 2016/S. Mt. Vernon, ME: Woods End Laboratories Inc; 2016. p. 3

48 Haney RL, Brinton WF, Evans E. Soil CO_2_ respiration: Comparison of chemical titration, CO_2_ IRGA analysis and the Solvita Gel System. Renew Agric Food Syst. 2008; 23: 171-176.

49 Haney RL, Franzluebbers AJ, Jin V, Johnson MV, Haney EB, White MV, et al. Soil organic C:N vs.water-extractable organic C:N. Open J Soil Sci. 2012; 2: 269-274.

50 Haney RL, Haney EB. Simple and rapid laboratory method for rewetting dry soil for incubations. Commun. Soil Sci. Plant Anal. 2010; 41:1493–1501.

51 Federer W, King F. Variations on split plot and split block experiment designs. John Wiley & Sons, Inc., New York; 2007. p. 219.

52 Bowley SR. A hitchhiker’s guide to statistics in biology. Generalized Linear Mixed Model Edition. Plants et al., Kincardine ON; 2015. ISBN 978-0-9685500-4-5.

53 Sainju UM, Schomberg HH, Singh BP, Whitehead WF, Tillman PG, Lachnicht-Weyers SL. Cover crop effect on soil carbon fractions under conservation tillage cotton. Soil Till Res. 2007; 96: 205–218.

54 Bationo A, Kihara J, Vanlauwe B, Waswa B, Kimetu J. Soil organic carbon dynamics, functions and management in West African agro-ecosystems. Agric Syst. 2007; 94:13–25.

55 Dinesh R, Suryanarayana MA, Chaudhuri SG, Sheeja TE, Shiva KN. Long-term effects of leguminous cover crops on biochemical and biological properties in the organic and mineral layers of soils of a coconut plantation. Eur J Soil Biol. 2006; 42:147–157.

56 Sainju UM, Senwo ZN, Nyakatawa EZ, Tazisong IA, Reddy KC. Soil carbon and nitrogen sequestration as affected by long-term tillage, cropping systems, and nitrogen fertilizer sources. Agric Ecosyst Environ. 2008; 127: 234–240.

57 Zhou X, Chengrong C, Wu H, Xu Z. Dynamics of soil extractable carbon and nitrogen under different cover crop residues. J Soils Sediments. 2012; 12, 844-853.

58 Puget P, Drinkwater, LE. Short-term dynamics of root- and shoot-derived carbon from a leguminous green manure. Soil Sci Soc Am J. 2001; 65: 771-779.

59 Schmidt MWI, Torn MS, Abiven S, Dittmar T, Guggenberger G, Janssens IA, et al. Persistence of soil organic matter as an ecosystem property. Nature. 2011; 478: 49-56.

60 Cortrufo MF, Wallenstein MD, Boot CM, Denef K, Paul E. The microbial efficiency-matrix stabilization (MEMS) framework integrates plant litter decomposition with soil organic matter stabilization: do labile plant inputs form stable soil organic matter? Glob Change Biol. 2013; 19: 988-995.

61 Bottner P, Sallih Z, Billes G. Root activity and carbon metabolism in soils. Biol Fert Soil. 1988; 7: 71-78.

62 Hui Z, Wenjun L, Xiaoming K, Xiaoyong C, Yanfen W, Haitao Z, et al. Changes in soil microbial community response to precipitation events in a semi-arid steppe of the Xilin River Basin, China. J Arid Land. 2019; 11: 97–110.

63 Sommerfeld RA, Moiser AR, Musselman RC. CO_2_, CH_4_ and N_2_O flux through Wyoming snowpack and implications for global budgets. Nature. 1993; 361: 140–142.

64 Linn DM, Doran JW. Effect of water filled pore space on carbon dioxide and nitrous oxide production in tilled and non-tilled soils. Soil Sci Soc Am J. 1984; 48: 1267–1272.

65 Six J, Frey SD, Thiet RK, Batten KM. Bacterial and fungal contributions to carbon sequestration in agroecosystems. Soil Sci Soc Am J. 2006; 70: 555-569.

66 Melero S, Panettieri M, Madejon E, Macpherson HG, Moreno F, Murillo JM, et al. Implementation of chiselling and mouldboard ploughing in soil after 8 years of no-till management in SW, Spain: Effect on soil quality. Soil Till Res. 2011; 112: 107-113.

67 Chen H, Hou R, Gong Y, Li H, Fan M, Kuzyakov Y. Effects of 11 years of conservation tillage on soil organic matter fractions in wheat monoculture in Loess Plateau of China. Soil Till Res. 2009; 106: 85-94.

68 Culman SW, Snapp SS, Freeman MA, Schipanski ME, Beniston J, Lal R, et al. Permanganate oxidizable carbon reflects a processed soil fraction that is sensitive to management. Soil Sci Soc Am J. 2012; 76: 494-504.

69 Prasad JVNS, Rao CS, Srinivas K, Jyothi CN, Venkateswarlu B, Ramachandrappa BK, et al. Effect of ten years of reduced tillage and recycling of organic matter on crop yields, soil organic carbon and its fractions in Alfisols of semi arid topics of southern India. Soil Till Res. 2016; 156: 131-139.

70 Stetson SJ, Osborne SL, Schumacher TE, Eynard A, Chilom G, Rice J, et al. Corn residue removal impact on topsoil organic carbon in a corn-soybean rotation. Soil Sci Soc Am J. 2012; 76: 1399-1406.

71 Martens DA. Plant residue biochemistry regulates soil carbon cycling and carbon sequestration. Soil Biol Biochem. 2000; 32:361–369.

72Martins MR, Angers DA. Different plant types for different soil ecosystem services. Geoderma. 2015; 237-238:266–269.

73 Wu Q-S, Cao M-Q, Zou Y-N, He X-H. Direct and indirect effects of glomalin, mycorrhizal hyphae, and roots on aggregate stability in rhizosphere of trifoliate orange. Sci Reports. 2014; 4: 5823.

74Hendrix PF, Chun-Ru, H, Groffman PM. Soil respiration in conventional and no-tillage agroecosystems under different winter cover crop rotations. Soil Till Res. 1988; 12: 135-148.

75 Mendes IDC, Bandick AK, Dick RP, Bottomley PJ. Microbial biomass and activities in soil aggregates affected by winter cover crops. Soil Sci Soc Am J. 1999; 63: 873-881.

76 Schmidt R, Gravuer K, Bossange AV, Mitchell J, Scow K. Long-term use of cover crops and no-till shift soil microbial community life strategies in agricultural soil. PLoS ONE. 2018; 13(2): e0192953.

77 Wang QR, Li Y, Klassen W. Changes of soil microbial biomass carbon and nitrogen with cover crops and irrigation in a tomato field. J Plant Nut. 2007; 30: 623-639.

78 Blanco-Canqui H, Ferguson RB, Jin VL, Schmer MR, Wienhold BJ, Tatarko J. Can cover crop and manure maintain soil properties after stover removal from irrigated no-till corn? Soil Sci Soc Am J. 2014; 78: 1368–1377.

79 Dignac MF, Derrien D, Barre P, Barot S, Cecillon L, Chenu C, et al. Increasing soil carbon storage: mechanisms, effects of agricultural practices and proxies.A review. Agron Sustain Dev. 2017; 37: 14 <https://doi.org/10.1007/s13593-017-0421-2>

80 Marschner B, Brodowski S, Dreves A, Gleixner G, Gude A, Grootes PM, et al. How relevant is recalcitrance for the stabilization of organic matter in soils? J Plant Nutr Soil Sci. 2008; 171:91–110.

81 Han P, Zhang W, Wang G, Sun W, Huang Y. Changes in soil organic carbon in croplands subjected to fertilizer management: a global meta-analysis. Nature Sci Reports. 2016; 6: 27199.

82Yue K, Peng Y, Peng C, Yang W, Peng X, Wu F. Stimulation of terrestrial ecosystem carbon storage by nitrogen addition: a meta-analysis. Sci Reports. 2016; 6:19895.

# **Supporting information**

**S1 Table. Impact of medium-term summer-planted cover crop (6-yrs) and crop residue management (retained (+R) or removed (-R) (3-yrs) on soil moisture gravimetric content in site-years 2015 and 2016.**

^a-c^ In each column, based on a protected LSD test, means followed by a different letter indicate statistical significant difference (*P*<0.05)

^z^ SE, standard error of mean

**S2 Table. Impact of medium-term summer-planted cover crops (6-yrs) (CC) on CC residue quality parameters (C concentration and C:N) during spring 2015 and 2016.**

^a-b^ For each parameter, based on a protected LSD test, means followed by a different letter indicate statistical significant difference (*P* < 0.05)

^z^ SE, standard error of mean

**S3** **Table. Impact of medium-term summer-planted cover crop (6-yrs) and crop residue retained (+R) or removed (-R) (3-yrs) on soil indicators from 0-15 cm depth in site-year 2015.**

^a-d^ For each indicator and effect, based on a protected LSD test, means followed by a different letter indicate statistical significant difference (*P*<0.05)

Cmin_2d_, cumulative 2d soil C mineralization; MBC, microbial biomass C; MBN, microbial biomass N; SLAN, Solvita labile amino N; OC, organic C; , total N; WAS, wet aggregate stability; WEOC, water extractable organic C; WEON, water extractable organic N

^z^ SE, standard error of mean

**S4 Table. Impact of medium-term summer-planted cover crop (6-yrs) and crop residue retained (+R) or removed (-R) (3-yrs) on soil indicators from 0-15 cm depth in site-year 2016.**

^a-c^ For each indicator and effect, based on a protected LSD test, means followed by a different letter indicate statistical significant difference (*P*<0.05)

Cmin_2d_, cumulative 2d soil C mineralization; MBC, microbial biomass C; MBN, microbial biomass N; SLAN, Solvita labile amino N; OC, organic C; WAS, wet aggregate stability; WEOC, water extractable organic C; WEON, water extractable organic N

^z^ SE, standard error of mean

**S5 Table. In a medium-term cover crop experiment, Spearman correlation coefficients between soil indicators sampled from 0-15 cm depth in 2015.**

*Significant correlation at *P* < 0.05

Cmin_2d_, cumulative 2d soil carbon mineralization; MBC, microbial biomass C; MBN, microbial biomass N; SLAN, Solvita labile amino N; OC, soil organic C; WAS, wet aggregate stability; WEOC, water extractable organic C; WEON, water extractable organic N

**S6 Table.** **In a medium-term cover crop experiment, Spearman correlation coefficients between soil indicators sampled from 0-15 cm depth in 2016.**

*Significant correlation at *P* < 0.05

Cmin_2d_, cumulative 2d soil carbon mineralization; MBC, microbial biomass C; MBN, microbial biomass N; SLAN, Solvita labile amino N; OC, soil organic C; WAS, wet aggregate stability; WEOC, water extractable organic C; WEON, water extractable organic N
