# Supplementary material for: Cover crop and crop residue removal effects on temporal dynamics of soil carbon and nitrogen in a temperate, humid climate
Source: PLoS One. 2020 Jul 9;15(7):e0235665. doi: 10.1371/journal.pone.0235665 (PMC7347225; doi:10.1371/journal.pone.0235665)
Supplement: Attachment — Submitted filename: Response to Editor R2. to submit.docx [file pone.0235665.s009.docx]

**Dear Editor-in-Chief,**

**Responses of authors to your comments are indicated in blue font.**

Comment 1: This is indeed expected. Labile compounds derive from decaying plant structures or exudates, so the chances of having a higher concentration at planting compared to the flowering stage is very low, unless planting is made on a stand already densely colonised by other plants. Please clarify it

Authors: Agreed. We have clarified this comment in the discussion section by inserting Line 350“As mentioned previously, tillage was conducted in mid May at both sites to incorporate the CC residues and prepare the soil for tomato transplanting. Approximately a week after tillage, tomato seedlings were transplanted. Cover crop residues, in addition to the root exudates and rhizodepositions released by the tomato roots undergo decay in soil over time and release labile compounds. Concentration of labile compounds is increased with an increase in plant density, size and development of root system. Therefore, greater concentration of rhizodeposits and labile fractions of C and N at the flowering than the planting stage is expected due to an increase in plant size at flowering (early to mid-July in our production region).”

Comment 2: Please revise throughout the text sentences like this. Soil indicator has no concentration. They are soil indicators, so they can be better or not, suitable or not. Each of that can have a concentration (e.g. mineral nitrogen).

The same occurs in the sentence “Overall, compared to no-CC and crop residue removal treatments, CCs and crop residue retention had greater concentrations of soil parameters temporally indicating the impact of soil C inputs on microbial processes with implications on soil quality.” and in other parts.

Authors: Agreed. We thoroughly reviewed the manuscript for phrase “indicator concentration” and have removed it from the manuscript (4 out of 4 instances). Additionally, for enhancing clarity, we have removed “soil indicators” at 19 instances in the manuscript and have replaced it with “labile and stable fractions of C and N”. Also, replaced “indicators” with “fractions” at 76 out of 78 instances.

Some examples of the revised sentences.

Line 30 “Medium-term CC usage enhanced the surface soil C and N storage.”

Line 32 “Overall, this study highlights the positive and synergistic influences of CCs and maintaining crop residues in increasing both labile and stable fractions of C and N and enhancing soil quality in a temperate humid climate.”

Line 254 “Larger pools of labile C and N with CC than no-CC across the season confirm the positive influences of CC on C and N dynamics in our medium-term experiment.”

Line 271 “Therefore, these results of larger C and N pools of both labile and stable fractions with +R than -R further indicates the potential of +R in increasing soil fertility, functionality, and C and N cycling.”

Line 389 “Moreover, early June (2WAT) represented the medium- (CC) and short- (residue incorporation) term effects on the tested indicators of soil quality. Likewise, Sainju et al. [7] reported increases in MBC, OC, and total N concentration following residue incorporation”

Line 402 “Results of larger pools of both stable and labile C and N with CC than no-CC at tomato harvest might be a reflection of the cumulative effects of CCing on soil build up and storage of C and N in our experiment.”

Line 456 “Overall, positive influence of CCs and crop residue retention on soil C and N storage compared to no-CC and crop residue removal treatments indicates the impact of soil C inputs on microbial processes with implications on soil quality.”

Comment 3 “In the previous version, just before this sentence there was the following sentence:

<< Peak period of tomato growth initiates in June and greater indicator concentration during June might have increased the nutrient availability to tomato roots and hence increased crop yield. >>

The rev_2 asked you to clarify it, whereas you deleted. I think it has a meaning, but I agree with rev_2 that such a meaning is not explained.

If you meant that tomato in your condition reaches its maximum growing rate in June, just explain it better and indicate that in this period it likely has the higher demand of nutrient per unit time and this helps you justifying the time of sampling

Authors: We have addressed this comment at line 393 “Additionally, it has been observed that soil sampling for assessing N status should be conducted when the crop is actively growing and has an increased N demand. In our production system, tomato plants start actively growing and increasing in size in June, thereby suggesting an increase in demand and N uptake by tomato roots. Thus, June represents a suitable time for soil sampling to understand N dynamics in our production system.” We did not include a comment about maximum or peak growth as it is confusing, and it is likely to occur later in the season. Instead, we added a sentence regarding the impact of cover crops on tomato yield at lines 406 “Furthermore, compared with the no-CC, CCs positively impacted the tomato primary productivity [33,36], further confirming the positive influences of CC on soil functionality and quality.”
